# The potential value of red blood cell distribution width in patients with invasive hydatidiform mole

**DOI:** 10.1002/jcla.22846

**Published:** 2019-03-18

**Authors:** Lingling Zhang, Youjun Xie, Lingling Zhan

**Affiliations:** ^1^ The First Affiliated Hospital of Guangxi Medical University Nanning China

**Keywords:** inflammatory parameters, invasive hydatidiform mole, neutrophil‐lymphocyte ratio, platelet‐lymphocyte ratio, red blood cell distribution width

## Abstract

**Background:**

Red blood cell distribution width (RDW) has attracted increasing attention in cancer. The aim of this study was to assess the changes of RDW in patients with invasive hydatidiform mole and analyze the relationship between RDW and invasive hydatidiform mole.

**Methods:**

A retrospective analysis was performed on 102 patients diagnosed as invasive hydatidiform mole in the First Affiliated Hospital of Guangxi Medical University from January 2009 to March 2018. A total of 120 healthy subjects were used as a control group. The Mann‐Whitney U test was used for comparison between the invasive hydatidiform mole and control groups. Comparison of RDW with other blood parameters was performed using Spearman's. The area under the ROC curve (AUC) and 95% confidence interval (95% CI) were also determined.

**Results:**

The RDW, platelet‐lymphocyte ratio (PLR), neutrophil‐lymphocyte ratio (NLR), and absolute lymphocyte count were significantly elevated in the invasive hydatidiform mole group compared with control group. The hemoglobin (Hb) concentration, mean red blood cell volume (MCV) and platelet count (PLT) were significantly lower in invasive hydatidiform mole group than control group. Grade III and above invasive hydatidiform mole patients had higher levels of RDW than grade I and II patients. Correlation analysis showed that RDW was negatively correlated with Hb, MCV, NLR, and neutrophil count, but positively correlated with PDW and different stages of invasive hydatidiform mole. The ROC curve showed that the AUC of the RDW was 0.660 (95% CI 0.581‐0.740; *P *< 0.01).

**Conclusion:**

This study reveals the potential value of RDW in invasive hydatidiform mole.

## 1 INTRODUCTION

Invasive hydatidiform mole is a malignant tumor originating from trophoblastic tissue. It is one of the gestational trophoblastic tumors that are characterized by invasive hydatid tissue into the myometrium or distant metastasis.^1^ Invasive hydatidiform moles occur in approximately 15% of patients with complete hydatidiform moles, while in other types of pregnancies it occurs less frequently. It may penetrate the myometrium to cause localized hemorrhagic necrosis.^2^ The prevalence of invasive hydatidiform mole has declined over the past 30 years due to economic and dietary improvements and a decline in overall birth rate. However, invasive hydatidiform mole has malignant tumor behavior and can undergo extensive metastasis.^3^ If left untreated it can be transformed into choriocarcinoma with poor prognosis, which can lead to significant morbidity and mortality.^4^ Therefore, early diagnosis of invasive hydatidiform mole is critical for rapid and accurate treatment management.

Red blood cell distribution width (RDW) is a parameter for routine examination of whole blood counts, which is a quantitative measure of changes in circulating red blood cell size.^5^ In addition to routine assessments in the differential diagnosis of anemia, studies have shown that there is a hierarchical independent relationship between high RDW levels and the occurrence of cervical, ovarian, and endometrial cancer.^6^, ^7^, ^8^ Similar to RDW, mean platelet volume, platelet‐to‐lymphocyte ratio, and neutrophil‐to‐lymphocyte ratio have also been reported as inflammatory markers in patients with inflammatory diseases, while they have prognostic significance for cancer.^9^, ^10^

It is currently believed that cancer is often the net result of chronic inflammation. Malignant tumors can also lead to malnutrition with chronic inflammation.^11^ In general, biomarkers (such as age, stage, and performance status) are used to stratify patients and guide treatment decisions. Since invasive hydatidiform mole does not have specific tumor markers, it is necessary to find markers for early detection and monitoring of high‐risk patients. At present, there are no data on the correlation between invasive hydatidiform mole and RDW. In this study, we intend to investigate the clinical significance of RDW in invasive hydatidiform mole.

## 2 PATIENTS AND METHODS

### 2.1 Patients

The study was approved by the Ethics Committee of the First Affiliated Hospital of Guangxi Medical University. Patients with a history of diabetes, cardiovascular disease, hepatitis B, chronic obstructive pulmonary disease, kidney disease, blood disease, or any medication or treatment that may interfere with hematology were excluded. A total of 102 inpatients diagnosed with invasive hydatidiform mole were enrolled between January 2009 and March 2018. A total of 120 healthy subjects were used as a control group. All patients with invasive hydatidiform mole were histologically and graded by pathology and/or cytology: 54 were stage I and II, 48 were stage III and IV.

### 2.2 Methods

A total of 2 mL venous blood sample was collected from each patient who was first diagnosed as an invasive hydatidiform mole and did not receive any treatment in the morning blood draw. All blood samples were placed in EDTA‐K2 anticoagulation tubes. All measurements were analyzed using a Beckman Coulter LH 780 Hematology Analyzer (Beckman Coulter, Brea, CA) within 30 minutes after blood collection. Where white blood cell count (WBC), hemoglobin (Hb) concentration, platelet count (PLT), mean red blood cell volume (MCV), mean platelet volume (MPV), RDW, platelet distribution width (PDW), absolute neutrophil count, (N) and absolute lymphocyte count (L) were obtained directly from the blood analyzer, while neutrophil‐lymphocyte ratio (NLR) and platelet‐lymphocyte ratio (PLR) were obtained by dividing absolute neutrophil count or platelet count by absolute lymphocyte count, respectively. The normal range for RDW in our hospital is 11.0%‐14.0%.

### 2.3 Statistical analysis

Statistical analysis was performed using SPSS 20.0 software (SPSS 20.0, Chicago, IL). The normality test was performed using the Kolmogorov‐Smirnov test, and the results showed that no measurement data conformed to the normal distribution. The test parameters of the non‐normal distribution are expressed as median (interquartile range). The Mann‐Whitney U test was used to analyze the parameters of invasive hydatidiform mole patients and control group. The association of RDW with other whole blood parameters was analyzed using Spearman's test in the invasive hydatidiform mole group. The area under the ROC curve (AUC) and 95% confidence interval (95% CI) were also determined. *P *< 0.05 was considered to be statistically significant.

## 3 RESULTS

According to exclusion criteria, a total of 102 patients (mean age 30.00 years; range 23.75‐37.00 years) with invasive hydatidiform mole were included in this study. A total of 120 normal people (mean age 31.50 years; range 26.00‐36.00 years) were used as a control group. WBC, Hb, MCV, PLT, MPV, PDW, absolute neutrophil and lymphocyte count, RDW, NLR, and PLR values were analyzed. Comparison of laboratory hematology parameters between groups is shown in Table 1. The results showed that the level of RDW, absolute lymphocyte count, NLR, and PLR was significantly higher in the invasive hydatidiform mole group compared with the control group (*P *< 0.05). The Hb concentration, MCV, and PLT were significantly lower in the invasive hydatidiform mole than the control group (*P *< 0.05).However, the age, WBC, MPV, PDW, and absolute neutrophil count between invasive hydatidiform mole and control groups were not significantly different (*P *> 0.05).

**Table 1:** Comparison of the parameters between the invasive hydatidiform mole and normal control groups

	Invasive hydatidiform mole group	Normal control group	*P*‐value
n	102	120	—
Age, y	30.00 (23.75‐37.00)	31.50 (26.00‐36.00)	0.294
WBC, × 10^9^/L	6.69 (5.64‐8.05)	7.00 (6.03‐8.03)	0.132
Hb, g/L	111.55 (100.58‐120.25)	146.00 (134.70‐153.90)	0.000
MCV, fL	86.46 (78.15‐90.45)	88.57 (86.20‐90.77)	0.000
PLT, × 10^9^/L	235.90 (202.03‐285.58)	259.35 (214.50‐295.43)	0.022
MPV, fL	8.43 (7.92‐9.10)	8.31 (7.80‐8.81)	0.212
PDW	0.16 (0.16‐0.17)	0.16 (0.16‐0.17)	0.992
N, × 10^9^/L	3.76 (3.03‐5.01)	3.67 (3.17‐4.52)	0.612
L,× 10^9^/L	1.93 (1.64‐2.34)	2.44 (1.97‐2.85)	0.000
RDW	0.16 (0.13‐0.16)	0.13 (0.13‐0.14)	0.000
NLR	1.91 (1.45‐2.52)	1.55 (1.25‐2.02)	0.000
PLR	120.63 (95.90‐151.78)	104.11 (84.46‐130.66)	0.003

Measurement data are expressed as median and quartile.

Hb, hemoglobin; L, absolute lymphocyte count; LR, neutrophil‐lymphocyte ratio; MCV, mean red blood cell volume; MPV, mean platelet volume; N, absolute neutrophil count; PDW, platelet distribution width; PLR, platelet‐to‐lymphocyte ratio; PLT, platelets; RDW, red cell distribution width; WBC, white blood cell count.

After pathological examination, 102 patients were diagnosed as invasive hydatidiform mole, of which 54 were stage I and stage II, and 48 cases were stage III and stage IV. The results showed that patients with grade III and IV invasive hydatidiform mole had higher RDW levels than patients with stage I and II [0.15(0.14‐0.16) vs 0.13(0.13‐0.15); *P *< 0.01] (Table 2). The ROC curve showed that the AUC of the RDW was 0.660 (95% CI 0.581‐0.740; *P *< 0.01).

**Table 2:** Comparison of hematology parameters levels according to the grade in invasive hydatidiform mole

	Grade I, II	Grade III, IV	*P*‐value
n	54	48	
RDW	0.13 (0.13‐0.15)	0.15 (0.14‐0.16)	0.000
Hb, g/L	112.50 (101.08‐121.00)	108.50 (98.08‐117.83)	0.139
MCV, fL	87.96 (83.29‐91.55)	83.65 (75.80‐88.35)	0.014
PLT, × 10^9^/L	245.35 (211.38‐290.38)	233.95 (201.13‐273.93)	0.535
L,× 10^9^/L	1.96 (1.69‐2.30)	1.87 (1.59‐2.41)	0.742
NLR	1.94 (1.54‐2.60)	1.83 (1.34‐2.29)	0.276
PLR	120.86 (95.45‐153.94)	119.3 (97.25‐150.08)	0.901

Measurement data are expressed as median and quartile.

Hb, hemoglobin; L, absolute lymphocyte count; MCV, mean red blood cell volume; NLR, neutrophil‐lymphocyte ratio; PLR, platelet‐to‐lymphocyte ratio; PLT, platelets; RDW, red cell distribution width.

**Figure 1:**
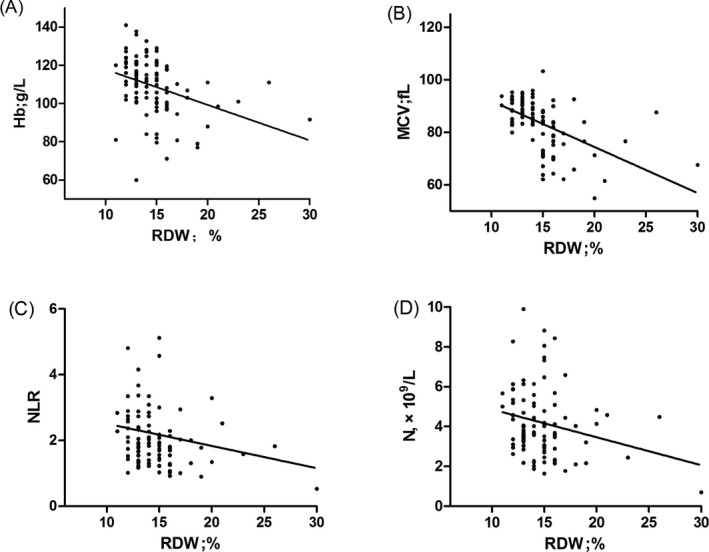
The correlation between RDW and (A) Hb, (B) MCV, (C) NLR and (D) N in patients with invasive hydatidiform mole. Hb, hemoglobin; MCV, mean red blood cell volume; N, absolute neutrophil count; NLR, neutrophil‐lymphocyte ratio; RDW, red cell distribution width

The association between RDW and other systemic blood inflammatory parameters was assessed using a correlation analysis. Statistical analysis showed RDW and Hb concentrations (*r* = −0.464; *P* = 0.000), RDW and MCV (*r* = −0.609; *P* = 0.000), RDW and NLR (*r* = −0.240; *P* = 0.015), RDW and absolute neutrophil counts (*r* = −0.221; *P* = 0.026) were negatively correlated. In addition, the study found a positive correlation between RDW and PDW (*r* = 0.230; *P* = 0.020), and RDW was positively correlated with clinical stage of invasive hydatidiform mole (*r* = 0.354; *P* = 0.000; Figure 1).

## 4 DISCUSSIONS

RDW is a parameter that reflects the heterogeneity of peripheral red blood cell volume and is considered to be a systemic inflammatory marker of in many chronic inflammation.^12^ As a low‐cost test, it is usually reported in the complete blood counts.^13^ Elevation of RDW may occur in ineffective red blood cell formation (iron deficiency, B12 or folate deficiency, and hemoglobinopathy), microangiopathic hemolytic anemias, increased red blood cell destruction (hemolysis) or post‐transfusion.^14^, ^15^ Pro‐inflammatory cytokines can inhibit erythropoietin‐induced erythrocyte maturation. Therefore, inflammation may cause the release of immature red blood cells into the peripheral circulation causing dysplastic changes in RBC formation.

To the best of our knowledge, this is the first study to investigate RDW in invasive hydatidiform mole. The results showed the RDW level of invasive hydatidiform mole group was significantly higher than the control group. Patients with grade III and above had significantly higher RDW levels than grade I and grade II invasive hydatidiform mole. In addition, we found that RDW was negatively correlated with Hb concentration, MCV, NLR, and absolute neutrophil count, but positively with PDW and different clinical stage of invasive hydatidiform mole.

Studies of different cancer types have established a link between inflammation and cancer. Inflammation plays a crucial role in the development of cancer. Cytokines and inflammatory mediators mediated by inflammatory factors in tumors may lead to the development, invasion, and metastasis of cancer, while RDW acts as an inflammatory factor during the onset of cancer. Similar to previous studies, RDW is elevated in many malignant tumors. Yang et al^16^ compared RDW values in 85 patients with colorectal cancer and 54 patients with colon polyps. RDW was observed to increase in colorectal cancer patients, and RDW was significantly different at each stage of colorectal cancer. In another study by Mehmet et al,^17^ it was found that lung cancer patients had significantly higher RDW and other blood inflammatory parameters than healthy subjects. Wan et al^18^ found that elevated levels of RDW in esophageal cancer. All patients enrolled were classified into high RDW group and low RDW group based on the detected RDW values. Median follow‐up was 21 months. In the univariate analysis, the high RDW group showed shorter disease‐free survival and unfavorable overall survival. Similarly, Huang et al^19^ retrospectively analyzed 203 breast cancer patients and divided the patients into two groups based on RDW values. It was found that high RDW was positively correlated with the clinical stage of cancer, while patients with elevated RDW had a poor prognosis. Qin et al^20^ found that RDW is increased in patients with ovarian cancer, and RDW levels increased with tumor stage changes. Kemal et al^8^ confirmed that 113 patients with endometrial cancer had higher RDW than 109 benign uterine lesions, and there was a correlation between cancer stage and RDW. It can be speculated that if RDW elevation is an indicator of inflammatory status, higher tumor grades, and more aggressive tumors usually cause systemic inflammation and lead to high levels of RDW. Due to the relationship between cancer and inflammation and the most important role of RDW in the inflammatory state, it is not surprising that high levels of RDW were positively correlated with the invasive hydatidiform mole stage in this study.

The findings of the present study supported the above study and confirmed a possible and critical relationship between inflammation and RDW. Literature studies have shown that RDW increases in inflammatory bowel disease, Hashimoto's thyroiditis,^21^ thyroid cancer,^22^ dermatomyositis, and polymyositis,^23^ indicating that RDW reflects the inflammatory state of the human body. Although recent studies have clearly demonstrated that RDW is a reliable biomarker for cardiovascular morbidity and mortality,^24^, ^25^, ^26^ the underlying mechanism between inflammation and elevated RDW levels remains unclear. It is currently believed that cancer is developed from chronic inflammation.^27^ The mechanism of RDW elevation in patients with invasive hydatidiform mole is still under investigation, but the possible mechanisms include the following. First, inflammatory factors can induce an increase in RDW, increase heterogeneity of peripheral red blood cell volume, and inhibit bone marrow hematopoietic function.^28^ The decrease in red blood cell survival rate, impaired iron metabolism and decreased red blood cell deformability can lead to inhibition of erythropoietin response at the same time. In patients with malignant tumors, overproduction of circulating cytokines such as interleukin‐6, tumor necrosis factor alpha and CRP has also been shown to play a key role in the induction of chronic inflammation.^29^, ^30^ In addition, many types of inflammatory factor receptors are detected on the surface of red blood cells, and it is speculated that red blood cells are involved in the inflammatory process. Hunziker et al^31^ found that inflammatory response can change red blood cell half‐life, leading to an increase in RDW. Second，patients with malignant tumors are usually accompanied by malnutrition. Gastrointestinal dysfunction and impaired immune function lead to malabsorption of nutrients such as iron, folic acid or VitB12, and can also be manifested as anemia, which causes an increase in RDW levels.^32^

This study has some limitations. First, this is a retrospective study of patients with invasive hydatidiform mole. The small sample size and lack of follow‐up prevent us from making a definitive conclusion about the relationship between RDW and invasive hydatidiform mole. Second, despite the adjustment of multiple risk factors and epidemics that affect RDW levels, there may be residual confounders and drugs not included in the analysis. In addition, we did not compare RDW values with other well‐known inflammatory parameters such as CRP, erythrocyte sedimentation rate (ESR), etc These indicators may help elucidate the mechanism by which RDW is elevated in patients with invasive hydatidiform mole. Therefore, in order to confirm our findings, large‐scale prospective studies are needed to investigate the importance of RDW in patients with invasive hydatidiform mole.

## 5 CONCLUSIONS

In conclusion, this study is the first to reveal the potential predictive role of RDW in patients with invasive hydatidiform mole. This clinical study also reported for the first time a significant correlation between clinical and pathological staging of patients with invasive hydatidiform mole. It is difficult to make appropriate monitoring plans and appropriate treatment strategies in time as the patient's clinical stage changes in clinical cancer treatment. Therefore, inflammatory parameters become important as prognostic or predictive markers. Since RDW is widely used for routinely implemented whole blood counts and is highly reproducible, this cheap and readily available parameter may serve as a new and convenient marker for understanding a patient's disease status and assessing tumor stage.

## AUTHOR CONTRIBUTIONS

The design and writing of this paper were completed by Lingling Zhang. Youjun Xie contributed to the collection of clinical data and analytical data. The manuscript was reviewed by Lingling Zhan.
